# Anthropology in the Clinic: The Problem of Cultural Competency and How to Fix It

**DOI:** 10.1371/journal.pmed.0030294

**Published:** 2006-10-24

**Authors:** Arthur Kleinman, Peter Benson

Cultural competency has become a fashionable term for clinicians and researchers. Yet no one can define this term precisely enough to operationalize it in clinical training and best practices.

It is clear that culture does matter in the clinic. Cultural factors are crucial to diagnosis, treatment, and care. They shape health-related beliefs, behaviors, and values [1,2]. But the large claims about the value of cultural competence for the art of professional care-giving around the world are simply not supported by robust evaluation research showing that systematic attention to culture really improves clinical services. This lack of evidence is a failure of outcome research to take culture seriously enough to routinely assess the cost-effectiveness of culturally informed therapeutic practices, not a lack of effort to introduce culturally informed strategies into clinical settings [3].

## Problems with the Idea of Cultural Competency

One major problem with the idea of cultural competency is that it suggests culture can be reduced to a technical skill for which clinicians can be trained to develop expertise [4]. This problem stems from how culture is defined in medicine, which contrasts strikingly with its current use in anthropology—the field in which the concept of culture originated [5–9]. Culture is often made synonymous with ethnicity, nationality, and language. For example, patients of a certain ethnicity—such as, the “Mexican patient”—are assumed to have a core set of beliefs about illness owing to fixed ethnic traits. Cultural competency becomes a series of “do's and don'ts” that define how to treat a patient of a given ethnic background [10]. The idea of isolated societies with shared cultural meanings would be rejected by anthropologists, today, since it leads to dangerous stereotyping—such as, “Chinese believe this,” “Japanese believe that,” and so on—as if entire societies or ethnic groups could be described by these simple slogans [11–13].

Another problem is that cultural factors are not always central to a case, and might actually hinder a more practical understanding of an episode (see Box 1).

**Box 1. Case Scenario: Cultural Assumptions May Hinder Practical Understanding**

A medical anthropologist is asked by a pediatrician in California to consult in the care of a Mexican man who is HIV positive. The man's wife had died of AIDS one year ago. He has a four-year-old son who is HIV positive, but he has not been bringing the child in regularly for care. The explanation given by the clinicians assumed that the problem turned on a radically different cultural understanding. What the anthropologist found, though, was to the contrary. This man had a near complete understanding of HIV/AIDS and its treatment—largely through the support of a local nonprofit organization aimed at supporting Mexican-American patients with HIV. However, he was a very-low-paid bus driver, often working late-night shifts, and he had no time to take his son to the clinic to receive care for him as regularly as his doctors requested. His failure to attend was not because of cultural differences, but rather his practical, socioeconomic situation. Talking with him and taking into account his “local world” were more useful than positing radically different Mexican health beliefs.

Historically in the health-care domain, culture referred almost solely to the domain of the patient and family. As seen in the case scenario in Box 1, we can also talk about the culture of the professional caregiver—including both the cultural background of the doctor, nurse, or social worker, and the culture of biomedicine itself—especially as it is expressed in institutions such as hospitals, clinics, and medical schools [14]. Indeed, the culture of biomedicine is now seen as key to the transmission of stigma, the incorporation and maintenance of racial bias in institutions, and the development of health disparities across minority groups [15–18].

## Culture Is Not Static

In anthropology today, culture is not seen as homogenous or static. Anthropologists emphasize that culture is not a single variable but rather comprises multiple variables, affecting all aspects of experience. Culture is inseparable from economic, political, religious, psychological, and biological conditions. Culture is a process through which ordinary activities and conditions take on an emotional tone and a moral meaning for participants.

Cultural processes include the embodiment of meaning in psychophysiological reactions [19], the development of interpersonal attachments [20], the serious performance of religious practices [21], common-sense interpretations [22], and the cultivation of collective and individual identity [23]. Cultural processes frequently differ within the same ethnic or social group because of differences in age cohort, gender, political association, class, religion, ethnicity, and even personality.

## The Importance of Ethnography

It is of course legitimate and highly desirable for clinicians to be sensitive to cultural difference, and to attempt to provide care that deals with cultural issues from an anthropological perspective. We believe that the optimal way to do this is to train clinicians in ethnography. “Ethnography” is the technical term used in anthropology for its core methodology. It refers to an anthropologist's description of what life is like in a “local world,” a specific setting in a society—usually one different from that of the anthropologist's world. Traditionally, the ethnographer visits a foreign country, learns the language, and, systematically, describes social patterns in a particular village, neighborhood, or network [24]. What sets this apart from other methods of social research is the importance placed on understanding the native's point of view [25]. The ethnographer practices an intensive and imaginative empathy for the experience of the natives—appreciating and humanly engaging with their foreignness [26], and understanding their religion, moral values, and everyday practices [27,28].

Ethnography is different than cultural competency. It eschews the “trait list approach” that understands culture as a set of already-known factors, such as “Chinese eat pork, Jews don't.” (Millions of Chinese are vegetarians or are Muslims who do not eat pork; some Jews, including the corresponding author of this paper, love pork.) Ethnography emphasizes engagement with others and with the practices that people undertake in their local worlds. It also emphasizes the ambivalence that many people feel as a result of being between worlds (for example, persons who identify as both African-American and Irish, Jewish and Christian, American and French) in a way that cultural competency does not. And ethnography eschews the technical mastery that the term “competency” suggests. Anthropologists and clinicians share a common belief—i.e., the primacy of experience [29–33]. The clinician, as an anthropologist of sorts, can empathize with the lived experience of the patient's illness, and try to understand the illness as the patient understands, feels, perceives, and responds to it.

**Box 2. The Explanatory Models Approach**

- What do you call this problem?
- What do you believe is the cause of this problem?
- What course do you expect it to take? How serious is it?
- What do you think this problem does inside your body?
- How does it affect your body and your mind?
- What do you most fear about this condition?
- What do you most fear about the treatment?

(Source: Chapter 15 in [38])

### The Explanatory Models Approach

One of us [AK] introduced the “explanatory models approach,” which is widely used in American medical schools today, as an interview technique (described below) that tries to understand how the social world affects and is affected by illness. Despite its influence, we've often witnessed misadventure when clinicians and clinical students use explanatory models. They materialize the models as a kind of substance or measurement (like hemoglobin, blood pressure, or X rays), and use it to end a conversation rather to start a conversation. The moment when the human experience of illness is recast into technical disease categories something crucial to the experience is lost because it was not validated as an appropriate clinical concern [34].

Rather, explanatory models ought to open clinicians to human communication and set their expert knowledge alongside (not over and above) the patient's own explanation and viewpoint. Using this approach, clinicians can perform a “mini-ethnography,” organized into a series of six steps. This is a revision of the Cultural Formulation included in the fourth edition of the Diagnostic and Statistical Manual of Mental Disorders (DSM-IV) (see Appendix I in [35]) [36,37].

### A Revised Cultural Formulation

#### Step 1: Ethnic identity.

The first step is to ask about ethnic identity and determine whether it matters for the patient—whether it is an important part of the patient's sense of self. As part of this inquiry, it is crucial to acknowledge and affirm a person's experience of ethnicity and illness. This is basic to any therapeutic interaction, and enables a respectful inquiry into the person's identity. The clinician can communicate a recognition that people live their ethnicity differently, that the experience of ethnicity is complicated but important, and that it bears significance in the health-care setting. Treating ethnicity as a matter of empirical evidence means that its salience depends on the situation. Ethnicity is not an abstract identity, as the DSM-IV cultural formulation implies, but a vital aspect of how life is lived. Its importance varies from case to case and depends on the person. It defines how people see themselves and their place within family, work, and social networks. Rather than assuming knowledge of the patient, which can lead to stereotyping, simply asking the patient about ethnicity and its salience is the best way to start.

#### Step 2: What is at stake?

The second step is to evaluate what is at stake as patients and their loved ones face an episode of illness. This evaluation may include close relationships, material resources, religious commitments, and even life itself. The question, “What is at stake?” can be asked by clinicians; the responses to this question will vary within and between ethnic groups, and will shed light on the moral lives of patients and their families.

#### Step 3: The illness narrative.

Step 3 is to reconstruct the patient's “illness narrative” [38]. This involves a series of questions (about one's explanatory model) aimed at acquiring an understanding of the meaning of illness (Box 2).

The patient and family's explanatory models can then be used to open up a conversation on cultural meanings that may hold serious implications for care. In this conversation, the clinician should be open to cultural differences in local worlds, and the patient should recognize that doctors do not fit a certain stereotype any more than they themselves do.

#### Step 4: Psychosocial stresses.

Step 4 is to consider the ongoing stresses and social supports that characterize people's lives. The clinician records the chief psychosocial problems associated with the illness and its treatment (such as family tensions, work problems, financial difficulties, and personal anxiety). For example, if the clinicians described in the case scenario in Box 1 had carried out step 4, they could have avoided the misunderstanding with their Mexican-American patient. The clinician can also list interventions to improve any of the patient's difficulties, such as professional therapy, self-treatment, family assistance, and alternative or complementary medicine.

#### Step 5: Influence of culture on clinical relationships.

Step 5 is to examine culture in terms of its influence on clinical relationships. Clinicians are grounded in the world of the patient, in their own personal network, and in the professional world of biomedicine and institutions. One crucial tool in ethnography is the critical self-reflection that comes from the unsettling but enlightening experience of being between social worlds (for example, the world of the researcher/doctor and the world of the patient/participant of ethnographic research). So, too, it is important to train clinicians to unpack the formative effect that the culture of biomedicine and institutions has on the most routine clinical practices—including bias, inappropriate and excessive use of advanced technology interventions, and, of course, stereotyping. Teaching practitioners to consider the effects of the culture of biomedicine is contrary to the view of the expert as authority and to the media's view that technical expertise is always the best answer. The statement “First do no harm by stereotyping” should appear on the walls of all clinics that cater to immigrant, refugee, and ethnic-minority populations. And yet since culture does not only apply to these groups, it ought to appear on the walls of all clinics.

#### Step 6: The problems of a cultural competency approach.

Finally, step 6 is to take into account the question of efficacy—namely, “Does this intervention actually work in particular cases?” There are also potential side-effects. Every intervention has potential unwanted effects, and this is also true of a culturalist approach. Perhaps the most serious side-effect of cultural competency is that attention to cultural difference can be interpreted by patients and families as intrusive, and might even contribute to a sense of being singled out and stigmatized [3,11,12]. Another danger is that overemphasis on cultural difference can lead to the mistaken idea that if we can only identify the cultural root of the problem, it can be resolved. The situation is usually much more complicated. For example, in her influential book, *The Spirit Catches You and You Fall Down*, Ann Fadiman shows that while inattention to culturally important factors creates havoc in the care of a young Hmong patient with epilepsy, once the cultural issues are addressed, there is still no easy resolution [33]. Instead, a whole new series of questions is raised.

### Determining What Is at Stake for the Patient

The case history in Box 3 gives an example of how simply using culturally appropriate terms to explain people's life stories helps the health professionals to restore a “broken” relationship and allows treatment to continue. This case is not settled, nor is it an example of any kind of technical competency. But there are two illuminating aspects of this case. First, it is important that health-care providers do not stigmatize or stereotype patients. This is a case study of an individual. Not all Chinese people fit this life story, and many contemporary Chinese now accept the diagnosis of depression. Second, culture is not just what patients have; clinicians also participate in cultural worlds. A physician too rigidly oriented around the classification system of biomedicine might find it unacceptable to use lay classifications for the treatment.

**Box 3. Case Scenario: The Importance of Using Culturally Appropriate Terms to Explain People's Life Stories**

Miss Lin is a 24-year-old exchange student from China in graduate school in the United States, where she developed symptoms of palpitations, shortness of breath, dizziness, fatigue, and headaches. A thorough medical work-up leaves the symptoms unexplained. A psychiatric consultant diagnoses a mixed depressive-anxiety disorder. Miss Lin is placed on antidepressants and does cognitive-behavioral psychotherapy, with symptoms getting better over a six-week period; but they do not disappear completely.

Subsequently, the patient drops out of treatment and refuses further contact with the medical system. Anthropological consultation discovers that Miss Lin comes from a Chinese family in Beijing—one of her cousins is hospitalized with chronic mental illness. So powerful is the stigma of that illness for this family that Miss Lin cannot conceive of the idea that she is suffering from a mental disorder, and refuses to deal with her American health-care providers because they use the terms “anxiety disorder” and “depressive disorder.” In this instance, she herself points out that in China the term that is used is neurasthenia or a stress-related condition. On the anthropologist's urging, clinicians reconnect with Miss Lin under this label.

For the late French moral philosopher Emmanuel Levinas, in the face of a person's suffering, the first ethical task is acknowledgement [39]. Face-to-face moral issues precede and take precedence over epistemological and cultural ones [40]. There is something more basic and more crucial than cultural competency in understanding the life of the patient, and this is the moral meaning of suffering—what is at stake for the patient; what the patient, at a deep level, stands to gain or lose. The explanatory models approach does not ask, for example, “What do Mexicans call this problem?” It asks, “What do you call this problem?” and thus a direct and immediate appeal is made to the patient as an individual, not as a representative of a group.

## Conclusion

What clinicians want to understand through the mini-ethnography is what really matters—what is really at stake for patients, their families, and, at times, their communities, and also what is at stake for themselves. If we were to reduce the six steps of culturally informed care to one activity that even the busiest clinician should be able to find time to do, it would be to routinely ask patients (and where appropriate family members) what matters most to them in the experience of illness and treatment. The clinicians can then use that crucial information in thinking through treatment decisions and negotiating with patients.

This is much different than cultural competency. Finding out what matters most to another person is not a technical skill. It is an elective affinity to the patient. This orientation becomes part of the practitioner's sense of self, and interpersonal skills become an important part of the practitioner's clinical resources [41]. It is what Franz Kafka said “a born doctor” has: “a hunger for people” [42]. And its main thrust is to focus on the patient as an individual, not a stereotype; as a human being facing danger and uncertainty, not merely a case; as an opportunity for the doctor to engage in an essential moral task, not an issue in cost-accounting [43].

## References

[pmed-0030294-b001] Kleinman A (2004). Culture and depression. N Engl J Med.

[pmed-0030294-b002] Kleinman A (1981). Patients and healers in the context of culture: An exploration of the borderland between anthropology, medicine, and psychiatry.

[pmed-0030294-b003] Kleinman A (2005). Culture and psychiatric diagnosis and treatment: What are the necessary therapeutic skills?.

[pmed-0030294-b004] DelVecchio Good M (1995). American medicine: The quest for competence.

[pmed-0030294-b005] Stocking GW (1996). Volksgeist as method and ethic: Essays on Boasian ethnography and the German anthropological tradition.

[pmed-0030294-b006] Abu-Lughod L, Fox RG (1991). Writing against culture.

[pmed-0030294-b007] Clifford J, Marcus GE (1986). Writing culture: The poetics and politics of ethnography: A School of American Research advanced seminar.

[pmed-0030294-b008] Gupta A, Ferguson J (1996). Culture, power, place: Explorations in critical anthropology.

[pmed-0030294-b009] Fischer MMJ (2003). Emergent forms of life and the anthropological voice.

[pmed-0030294-b010] Betancourt JR (2004). Cultural competence—Marginal or mainstream movement?. N Eng J Med.

[pmed-0030294-b011] Taylor J (2003). The story catches you and you fall down: Tragedy, ethnography, and “cultural competence. Med Anthropol Q.

[pmed-0030294-b012] Lee SA, Farrell M (2006). Is cultural competency a backdoor to racism?. Anthropology News.

[pmed-0030294-b013] Green JW (2006). On cultural competence. Anthropology News.

[pmed-0030294-b014] Taylor J (2003). Confronting “culture” in medicine's “culture of no culture. Acad Med.

[pmed-0030294-b015] Lee S, Lee M, Chiu M, Kleinman A (2005). Experience of social stigma by people with schizophrenia in Hong Kong. Br J Psychiatry.

[pmed-0030294-b016] Keusch GT, Wilentz J, Kleinman A (2006). Stigma and global health: Developing a research agenda. Lancet.

[pmed-0030294-b017] Wailoo K (2001). Dying in the city of the blues: Sickle cell anemia and the politics of race and health.

[pmed-0030294-b018] United States Department of Health and Human Services [HHS] (1999). Mental health: A report of the Surgeon General. http://www.mentalhealth.samhsa.gov/cmhs/surgeongeneral/surgeongeneralrpt.as.

[pmed-0030294-b019] Moerman DE, Guess HA, Kleinman A, Kusek JW, Engel LW (2002). Explanatory mechanisms for placebo effects: Cultural influences and the meaning response.

[pmed-0030294-b020] Goffman E (1959). The presentation of self in everyday life.

[pmed-0030294-b021] Barth F (1987). Cosmologies in the making: A generative approach to cultural variation in Inner New Guinea.

[pmed-0030294-b022] Sahlins M (1978). Culture and practical reason.

[pmed-0030294-b023] Holland D, Lachicotte W, Skinner D, Cain C (1996). Identity and agency in cultural worlds.

[pmed-0030294-b024] Kleinman A (1999). Moral experience and ethical reflection: Can medical anthropology reconcile them?. Daedalus.

[pmed-0030294-b025] Geertz C (1983). Local knowledge.

[pmed-0030294-b026] Jackson M (1996). Things as they are.

[pmed-0030294-b027] Geertz C (1972). The interpretation of cultures.

[pmed-0030294-b028] Marcus G, Fischer MMJ (1986). Anthropology as cultural critique.

[pmed-0030294-b029] Slobodin R (1997). W. H. R. Rivers: Pioneer anthropologist, psychiatrist of the ghost road.

[pmed-0030294-b030] Barker P (1991). Regeneration.

[pmed-0030294-b031] Sacks O (1996). An anthropologist on Mars.

[pmed-0030294-b032] Konner M (1988). Becoming a doctor.

[pmed-0030294-b033] Fadiman A (1998). The spirit catches you and you fall down.

[pmed-0030294-b034] Kleinman A, Benson P (2004). La vida moral de los que sufren de la enfermedad y el fracaso existencial de la medicina. Monografías Humanitas.

[pmed-0030294-b035] [Anonymous] (1994). Diagnostic and statistical manual of mental disorders. http://www.psychiatryonline.com/resourceTOC.aspx?resourceID=1.

[pmed-0030294-b036] Novins DK, Bechtold DW, Sack WH, Thompson J, Carter DR (1997). The DSM-IV outline for cultural formulation: A critical demonstration with American Indian children. J Am Acad Child Adolesc Psychiatry.

[pmed-0030294-b037] Mezzich JE, Kirmayer LJ, Kleinman A, Fabrega H, Parron DL (1999). The place of culture in DSM-IV. J Nerv Ment Dis.

[pmed-0030294-b038] Kleinman A (1988). The illness narratives: Suffering, healing, and the human condition.

[pmed-0030294-b039] Levinas E, Smith MB, Harshav B (2000). Useless suffering.

[pmed-0030294-b040] Levinas E (1998). Otherwise than being: Or beyond essence.

[pmed-0030294-b041] Goethe (1978). Elective affinities.

[pmed-0030294-b042] Lensing LA (2003 February 28). Franz would be with us here. Times Literary Supplement.

[pmed-0030294-b043] Kleinman A (2006). What really matters: Living a moral life amidst uncertainty and danger.

